# Overcoming barriers to implementation of patient engagement in clinical trials: feasibility testing of an embedded study

**DOI:** 10.1186/s40900-025-00689-0

**Published:** 2025-02-26

**Authors:** Geneviève Castonguay, Sylvain Bédard, Anick Dubois, Émilie Lessard, Léna Rivard, Ghislaine Rouly, Antoine Boivin

**Affiliations:** 1 Canada Research Chair in Partnership with Patients and Communities, Montreal University Hospital Research Center, 850 St-Denis St, Montreal, QC H2X 0A9 Canada; 2 Center of Excellence for Partnership with Patients and the Public, Montreal University Hospital Research Center, 850 St-Denis St, Montreal, QC H2X 0A9 Canada; 3 Montreal Heart Institute, 5000 Bélanger St, Montreal, Québec H1T 1C8 Canada; 4 Department of Family Medicine, Université de Montréal, Pavillon Roger-Gaudry, 2900 chemin de la Tour, Montreal, QC Canada

**Keywords:** Patient Engagement, Patient involvement, Clinical trial, Feasibility, Barriers, Embedded study, Implementation

## Abstract

**Background:**

Patient engagement is attracting considerable interest as a potential strategy to improve the conduct of clinical trials, with evidence of significant improvement in research participant recruitment. However, impact on the retention and adherence of clinical trial participants requires further studies. Embedded studies are specific research designs where a secondary study is “embedded” into a larger host study. We aimed to investigate the feasibility of embedding a study of patient partnership in research, within an ongoing multi-center clinical trial on drug treatment.

**Methods:**

We developed and embedded a patient engagement intervention (embedded study) into a phase 3 randomized clinical drug trial (host study). The patient engagement intervention consisted of discussions between host study participants and a patient partner, to improve research participants’ experience and retention in the clinical trial. We carried out individual semi-structured interviews with patient partners and other research team members involved in the development and implementation of the embedded study, as well as an analysis of project documents. Data were analyzed using qualitative thematic analysis.

**Results:**

Factors impacting feasibility and lessons learned for future embedded studies on engagement science were identified. Barriers that curtailed the implementation of patient engagement into an ongoing clinical trial included: the late integration of the embedded study into the host clinical trial, different visions of patient partnership and its potential benefits, differences in communication style and preferences, a lack of fit between the specific needs of the host study and the proposed engagement model, and an overall sense of burden. Integrating patient partners into the host clinical trial was seen as potentially beneficial in improving the experience of participants in the host clinical trial through experience sharing, providing support for the consent process, and improving knowledge transfer.

**Conclusions:**

This feasibility study offers insights into how contextual factors and decisions made during the design phase can impact the implementation of patient engagement studies embedded in a clinical trial. Findings suggest that knowledge of the clinical trial context (e.g., organizational, administrative, regulatory, ethics) and early collaboration among embedded study and host study teams before initiation of both studies are key conditions for success.

**Supplementary Information:**

The online version contains supplementary material available at 10.1186/s40900-025-00689-0.

## Abstract (plain language summary)

Clinical studies aim to find out if a new treatment or intervention works well. For the results of these studies to be trustworthy, it is important to enlist many participants, and for these participants to remain in the study until it is over. Studies show that involving patients as partners in clinical studies can help enlist participants.

This study aimed at exploring the feasibility of creating a collaboration between research nurses and patient partners to enlist and keep clinical study participants.

Two teams, one working on patients in research and another conducting a clinical drug study, worked together on a detailed research plan.

Through interviews and documents used in the study, we learned about the challenges of including a patient partner into the clinical study team. For example, it is difficult to include a project into a clinical study that is already underway, when the teams working together have different visions of patient engagement in research, when these teams use different ways of communicating, and when the intervention offered to participants does not meet their needs well. However, patient partners could improve participants’ experience by sharing their experiences and providing information about the study and its results.

We have drawn several important lessons from this study. The key conditions for success seem to be: knowing the context of the clinical study, like the organization’s culture, working to build a partnership between the teams involved in the project, and working together early in the research process.

**Supplementary Information:**

The online version contains supplementary material available at 10.1186/s40900-025-00689-0.

## Background

Patient engagement in research is gaining considerable interest as a strategy to improve the conduct of clinical trials. Clinical trials have shown that patient engagement can significantly improve participant recruitment, although its impact on the retention and adherence of participants requires further investigation [1]. One innovative approach to studying patient engagement is through studies embedded within larger ‘host’ clinical trials. In this design, the host clinical trial addresses the primary research question (e.g., evaluating the efficacy of a clinical drug), while the embedded study explores a secondary research question (e.g., assessing the impact of patient engagement on recruitment and retention). Embedded studies enable the exploration of patient engagement interventions by leveraging the infrastructure of the host clinical trial. For example, they have been used successfully to evaluate how patient engagement affects consent and recruitment processes in clinical trials [1–3].

Studies embedded in clinical trials have several advantages, including rigorous testing of specific interventions or processes within the context of a host study. From an epistemological perspective, embedded studies enable the development of credible evidence across diverse paradigms, including positivist, constructivist, and pragmatic approaches, depending on the focus of the study. However, the operationalization of embedded studies raises many feasibility issues, including increased complexity, necessity for coordination and alignment between the host and embedded study teams, and acceptability of the engagement intervention for study participants and research professionals [4].

To evaluate the practicality of implementing a patient engagement embedded study within a host clinical trial, we conducted a feasibility study. Such studies provide specific information needed to plan subsequent research, including identifying aspects of the study design that require refinement [5]. Feasibility studies assess the practicality of study designs, measures, procedures, recruitment criteria, and operational strategies to be considered in a subsequent study [6]. This particular feasibility study also enabled the evaluation of issues encountered when integrating a study into a host clinical trial that had already begun before the embedded study was launched.

### Objectives

This paper aims to contribute to the science of engaging patients in clinical trials by addressing the following research question: to what extent is it feasible to conduct a randomized trial of patient engagement embedded into an ongoing multi-center clinical trial? Specific objectives of the feasibility study were structured according to the classification proposed by Thabane et al. (2010) [5]:

1. Assess the acceptability of a patient engagement intervention at the levels of study sites, research professionals and study participants;
2. Determine the feasibility of collecting and exchanging process and outcome data between the host and embedded study teams;
3. Determine recruitment and retention rates for the engagement intervention to assess the feasibility of conducting a definitive clinical trial and calculate the sample size.

This paper reports on the feasibility of conducting such a project and lessons learned for future embedded studies in engagement science, drawing from analyses of interviews and study documents.

## Methods

### Design

Feasibility study for a randomized trial of patient engagement, embedded in a host clinical trial.

### Host study

The host clinical trial was a large-scale randomized phase 3 multicenter clinical trial assessing the use of a marketed drug as a preventive treatment in adults with a known health condition. The clinical trial was led and sponsored by an academic health research institution in Canada and was funded through public grants and philanthropy. The host study was already underway for several years when the idea of embedding a patient engagement study was proposed, therefore all aspects of study design (e.g., ethics approval and consent forms, job descriptions and staff recruitment, data collection and sharing procedures, inter-institutional contracts and budgets) were already established. In the year that funding for the patient engagement embedded study was obtained, a genetic study was also integrated into the host clinical trial.

Clinical researchers involved in the host study approached a team with expertise in patient engagement, including patient partners with research experience, a researcher in the area of patient engagement, research personnel and managers, to conduct a patient engagement study. This initiative was suggested by senior researchers of the clinical trial. The host study team was also motivated by a range of reasons, including the desire to improve recruitment and retention of participants in the clinical trial, promote patient wellbeing and be innovative. Researchers from both teams included a patient partnership component in a larger proposal from the host institution and jointly obtained public funding (4-year grant). Both teams envisioned the feasibility study as a preparatory step for a larger study that would be conducted later (specifically, a step-wedge cluster randomized trial of patient engagement embedded into a host clinical trial).

### Embedded study

The embedded study team proposed a patient engagement intervention aimed at supporting host clinical trial research participants’ informed recruitment, experience and retention in the clinical trial. In discussions with the host research team, patient partners were defined as someone with the condition targeted by the clinical trial and, if possible, with experience as a participant in the clinical trial. The blueprint for the engagement intervention took a partnership orientation: patient partners would work collaboratively with research nurses in building ongoing relationships with clinical trial participants, including one-to-one meetings between study participants and the patient partner, during which they would discuss the experience of participating in the clinical trial and potential questions and issues regarding their participation. Partnership is an engagement relationship characterized by power sharing and coleadership [7]. This partnership orientation extended to the embedded study team, which was co-led by an experienced patient partner (SB), with input from a senior patient mentor who acted as advisor on the project (GR). To consult the GRIPP2 (Guidance for Reporting Involvement of Patients and the Public) for this paper, please see Additional file 1.

### Data collection

It was initially planned that data collection for the feasibility study would draw extensively from research data being collected as part of the host study to minimize burden on study participants and research professionals. This planned data collection included quality of life questionnaire scores, participant-reported assessment of medication use, enrollment and retention rates.

The feasibility and perceived acceptability of the embedded study were documented using individual interviews (*n* = 8) with patient partners, a research nurse, study managers and principal investigators (Table 1).

**Table 1:** Roles of the interviewees (*n* = 8) within the study

Host study team (*n* = 4)	Embedded study team (*n* = 4)
Principal investigator (host study) Patient partner recruited for the study Research nurse Clinical project manager	Principal investigator (embedded study) Patient partner mentor Engagement intervention coordinator Engagement research coordinator

A member of the embedded study team (EL) who was not involved in the clinical trial nor the embedded study (an anthropologist with extensive experience in qualitative research) conducted semi-structured interviews using an interview guide. This guide, inspired by Sekhon et al.’s [8] theoretical framework of acceptability, was developed to examine the roles of each stakeholder group in the study, factors influencing the integration of the engagement component into the clinical trial, as well as challenges and impacts of this integration from the perspective of each interviewee. Interviews were conducted by videoconference via Zoom, lasted on average 52 min (26–86), were audio-recorded with the consent of interviewees, and transcribed verbatim.

The embedded study team collected study implementation logs to track issues arising throughout the project and modifications made to the research protocol. In addition, the embedded study team planned to collect data on costs and activities using tracker sheets.

### Analysis

We conducted a protocol analysis to document the changes resulting from interactions between the host and embedded study teams. These interactions occurred mainly during the preparation of a detailed protocol for submission to the host institution’s ethics committee, after obtaining 4-year funding for the study. We referred to the study implementation logs, which documented each interaction between the teams, to explain the changes. We also conducted an interview analysis, which provides the perspectives of stakeholders who were involved in this process. This combined analysis is key to understanding the feasibility, barriers, benefits and lessons learned from embedding a patient engagement study into an ongoing large-scale multicenter randomized clinical trial, as well as the adaptations made throughout the implementation process.

The researcher (EL) who conducted the individual interviews also led their thematic analysis based on the steps described by Braun & Clarke [9]: data familiarization, generating codes, searching for themes, reviewing and defining themes, and reporting findings. Using QSR NVivo 12, transcripts were analyzed inductively, constructing codes from the data. The analysis of changes in the study protocol consisted of a systematic comparison of the protocol proposed by the embedded study team and the final approved protocol, section by section, documenting similarities and differences between documents in a table. The origin of changes between the two versions was also noted.

Once each data set had been analyzed independently, we merged the results to draw conclusions [10]. This approach enabled us not only to gather rich data on the integration of a patient engagement study embedded into an ongoing multicenter randomized clinical trial, but also to examine the feasibility, barriers, benefits and lessons learned from such an endeavour from the perspective of both the host and embedded study teams.

### Ethical approval

The Institutional Review Boards of the two institutions involved (host and embedded study teams) approved the embedded study (# MP- 33-2014-1559; # 20.025).

## Results

The protocol analysis resulted in a 22-page table with detailed notes on the similarities and differences between the protocol proposed by the embedded study team and the final approved protocol. The results revealed a large number of changes in both substantive content and emphasis made over five months of intensive work between teams.

In total, eight interviews were conducted with members of the host and embedded study teams who were involved in the development and implementation of the engagement embedded study. This included: the principal clinical investigator of the host study, a research nurse, the clinical project manager, the principal investigator of the engagement component, the engagement research coordinator, the engagement intervention coordinator, a patient partner mentor and the patient partner recruited for the embedded study. The consent forms signed by interviewees specified that data may be published in a way that would not identify participants. Therefore, we have replaced all the names of the interviewees with pseudonyms. Results will be presented according to the aims of this study (feasibility, barriers, benefits and lessons learned).

### Evolution in design of the embedded study over time

It is worth noting that the host study team had initially approached the embedded study team with a different research proposal. The central idea of this early proposal was to assemble one group of patient partners to carry out 2–3 focus groups with members of the clinical trial and genetic study teams, as well as research participants. This group would create knowledge transfer tools and a patient partnership strategy for the clinical trial to optimize recruitment and retention. This proposal was designed to have minimal impact in terms of resources (e.g., clinical research nurses’ time) and organisational structure of the ongoing clinical trial. Key considerations for the host study team at this stage were that a genetic study had just been initiated in the clinical trial and that no funding had been allocated to the clinical team to manage the engagement study.

The patient engagement intervention (embedded study described above) proposed by the engagement research team had a broader scope of engagement and was expected to have more impact on research participants, as well as implications for the field of engagement science. This proposal was developed into a full protocol by the engagement research team. When systematically comparing this protocol and the final approved protocol, the documented differences reflect, first, a reduction in the scope of the engagement component of the study. Whereas the protocol proposed by the embedded study team described a randomized trial embedded in a multisite study, the later version presented a single-site embedded study that could be extended to other sites later. This limitation of the engagement component to one of several clinical trial sites meant that randomization was not possible. Similarly, although the inclusion and exclusion criteria remained unchanged, the potential pool of study participants was reduced. For the host study team, the embedded study team’s protocol proposed a much broader scope of intervention and required significantly more resources and commitment than anticipated. In their view, it was not feasible to implement as proposed given the context of the ongoing clinical trial. However, the first site chosen to implement the intervention had the largest number of recruited participants for the host study.

The duration of the engagement intervention for each participant, planned for 18 months in the protocol proposed by the embedded study team, was shortened by 6 months. This was necessary because of the time needed to agree on protocol modifications, receive ethics approvals from both institutional ethics committees and complete the study with only 2 years remaining of the granting period (for funding obtained jointly by the host and embedded teams).

In addition, the integration of the engagement intervention into the clinical trial was reduced. The protocol proposed by the embedded study team presented an engagement dyad formed by a patient partner working together with a clinical research nurse to recruit and support the embedded study participants throughout the clinical trial. In the final protocol, no interaction was planned between the nurse and patient partner, who was described as an “additional resource for participant follow-up”. The host clinical trial team requested these changes due to organisational issues and limited resources. For example, the first protocol would have required coordinating the schedules of all parties (patient partner, clinical research nurse, and participant) so that they would be present on-site during participants’ follow-up visits. There would also have been complexity in having the patient partner present at the clinical site when a patient is recruited as a host study participant, as it may be anytime during the day.

Thus, the final protocol followed the standard recruitment method at the clinical research institution: clinical trial participants would receive a brochure from the research nurses introducing the engagement intervention and the contact information of the engagement research team. Those interested in participating in this optional embedded study would need to initiate contact with the embedded study team by phone or email. They could speak with the patient partner before deciding to participate. The consent procedure was verbal to facilitate recruitment. The host research team required this recruitment method due to limited resources. Specifically, clinical nurses already had two studies (main study and genetic study) and consent forms to present, and presenting a third consent form for the embedded study would have required more of their and the clinical trial participants’ time. In addition, the clinical research institution had no dedicated funds to support the embedded study. Moreover, this was the first time patient partners were included in a clinical trial in such a role, and all stakeholders (clinical team, patients, research participants, ethics committees) needed to be educated on new concepts related to patient engagement.

Another difference between the protocols concerned changes in the description of outcomes and data collection procedures. In the initial protocol, this description focused on the various stakeholders and used primary data already being collected for the host clinical trial (e.g., recruitment and retention rates). In the final protocol, a detailed description of tools and procedures specific to the embedded study was provided, and outcomes based on data from the main study at 6, 12, and 18 months no longer appeared (only data from questionnaires administered at baseline and at the end of the study). Among the factors that contributed to these changes, a contract research organization (third party) managed the study data so that obtaining it for the embedded study would have entailed additional resources, and the consent of the participants was necessary to share their nominative data with another research establishment/researcher (the embedded study team). Thus, in the final protocol, the sharing of non-nominative data remained unchanged (e.g. recruitment and retention rates). The available nominative data (quality of life questionnaires at the beginning and end of the main study) would be transferred to the engagement team at the end of the embedded study if participants consented. As will be described later, due to restrictions on recruitment and in-person visits at clinical institutions imposed by provincial health authorities for more than a year during the pandemic, no participant was recruited for the embedded study. Therefore, data collection and transfer could not be carried out as agreed between teams.

In addition to changes related to logistical limitations, other changes to the protocol pertained to professional, institutional and scientific cultures. While the host study team operated in an institutional and professional culture requiring detailed pre-planned research procedures to ensure the scientific integrity of the clinical drug trial, the embedded study team operated within a scientific culture characterized by participatory approaches and constructivist epistemologies, which view research as a flexible process of discovery and knowledge co-creation. The embedded study team had planned that intervention modalities would be co-designed by both teams, including the patient partner and clinical research nurse dyad, in the early phases of the embedded study. However, the host research team did not wish to engage in this open-ended process and judged that the protocol would only get ethical approval with predetermined details of the engagement intervention. In their experience, all interactions with research participants had to be described to ensure compliance with ethical principles. Before submission, the host study team consulted with their ethics committee, which oversaw the clinical trial, to inform them of the embedded study and receive advice on what should be described in the protocol. This was the first time that the host study team sought ethical approval to include patient partners to support research participants’ follow-up. It was important for them to ensure that the additional embedded study would not impact the conduct of the ongoing host clinical trial, which had to follow strict regulatory and ethical rules. In the end, all details of the engagement intervention were predetermined between both teams and described in the protocol submitted for ethical approval. The final protocol also put greater emphasis on the exploratory/pilot nature of the embedded study, the mixed methods design, and the teams.

During the review process, the ethics committee overseeing the clinical trial requested further changes to the engagement study protocol. Notably, the roles and responsibilities of the research teams (engagement and clinical) and the collaboration between them at different stages of the study were specified. Further details were also provided for the ethics committee on the intervention, including who would initiate the exchanges between the participants and patient partner, where and for how long. Several additions concerned specifically the patient partner: their status as a member of the research team, details on the confidentiality agreement that they would sign, the basis for their remuneration and compensation for expenses, and that they would not be conducting research interviews. Additional information was also provided on the terms and conditions for the participation of research team members in interviews.

### Feasibility, barriers and perceived benefits for embedding an engagement study

*Perceived potential benefits*. Interviewees identified several potential benefits of integrating patient partners into the host study. The first was to improve the experience and support of host study participants during the clinical trial through experience sharing.

“It’s really about accompanying research participants on issues of an experiential nature. So the experience of being part of the clinical trial in question. For example, can I go on a trip? What do I do if I want to go on a trip?” Martin.

Patient partners could also provide support in the consent process, including a better understanding of the issues, free and informed consent and increased recruitment.

“What I understood was that I was there as an external support to answer questions […] within the research team as a person who had lived, who had participated, […] to bring the participants’ point of view. […] Because I’ve been, I’ve participated in the study, well, my experience could perhaps enable other people at least to make a decision about whether or not to participate.” Henry.

Finally, some interviewees mentioned that patient partners could improve knowledge transfer for the host clinical trial. For example, patient partners could keep host clinical trial participants regularly informed (i.e., by newsletter), promoting a sense of belonging among study participants, thereby improving their experience and retention in the host clinical trial.

*Feasibility*. While integrating patient partners in a clinical trial was seen as potentially beneficial by many interviewees, embedding such a study in practice faced many feasibility issues. The first issue was the acceptability of the patient engagement intervention for some of the stakeholders.

Interviewees reported that clinical research nurses had expressed an initial reluctance regarding the role and added value of the patient partner. For these nurses, patient partners were perceived as a threat to their professional identity, as they considered they were already fulfilling the role of listening and supporting clinical trial participants that was proposed for patient partners. One interviewee also mentioned that the first patient partner recruited for the study was a former participant in the clinical trial, and that a research nurse had expressed a reluctance to work with this patient as a research partner rather than as a study participant or regular clinic patient.

Interviewees noted that the acceptability of the intervention increased following the patient engagement training, when the role of the patient partner was clearly articulated around sharing lived experience and the potential benefits on recruitment and retention rates.

A reluctance to include a patient partner in the early phases of research, including clinical trial protocol design and consent form development, was also reported by some host study team interviewees. Clinical researchers expressed the need to see evidence of the benefits of including a patient partner, even if the inclusion of patient partners was a focus of research inquiry. As one member of the host study team explained, statisticians and other clinical researchers wondered what the benefit was for them to have a patient partner read the protocol, which they saw as an additional step in the review process.

An interviewee from the host study team mentioned that acceptability was very high among study participants, and the reception was enthusiastic when recruitment started. However, the pandemic halted recruitment and no host study participants were ultimately recruited for the embedded study.

Interview results suggested that the absence of enrollment into the embedded study was explained not only by the short recruitment period, but also by the nature of the host clinical trial, which tested the preventive effects of a commercialized drug with little or no side effects. One of the interviewees reasoned that the greater the symptoms of a disease, the limitations of a condition, or the side effects of treatment, the more relevant and important the role of the patient partner may be in supporting research participants. In this case, there was little or no need for support compared with a clinical trial aimed at prolonging life or curing a potentially fatal disease.

“Maybe one last factor that […] I learned along the way was the nature of the health problem we were dealing with in the primary study, that probably contributed to the fact that there may not have been […] as much of a need to have a patient partner.” Peter.

*Barriers*. The context of the host study was identified as the main barrier to the development and implementation of the patient engagement embedded study. The embedded study was seen by some interviewees as imposed on the host research team by senior researchers of the host trial. At the time, the host study protocol and consent forms had been designed and approved by all relevant authorities and recruitment had been ongoing for years. However, recruitment into the host study proved difficult for multiple reasons and the team was under great pressure to recruit research participants. For example, medical patients were reluctant to participate in the host study because it required a long follow-up period with multiple mandatory visits to the clinical site, including a 90-minute screening visit. In addition, another study (genetic) was already attached to the host study, which meant that clinical nurses presented two consent forms to potential research participants. When recruitment began for the embedded engagement study, a third consent form was added. The clinical nurses considered that they did not have the time to present all three studies in a single visit and could not ask participants for more time during their screening or follow-up visits.

From the host study team’s perspective, decisions to modify the protocol were based on how to overcome contextual barriers to integrating the patient partner component into the host study. Specifically, many changes were due to a lack of resources in the clinical team (e.g., limited staff and funding, high staff turnover) to support the engagement component. For example, due to the nurses’ limited time, they felt it was impossible for them to work with a patient partner in an engagement dyad. A member of the host study team also reported that the engagement study was more difficult to carry out because no member of their team was dedicated to this embedded study.

The COVID-19 pandemic also contributed to the feasibility outcomes. At the start of the pandemic, no recruitment was possible, as health authorities did not permit in-person visits for clinical trials conducted in health institutions. Later, the main study shifted to remote recruitment, but yielded a very low recruitment rate and recruitment for the embedded study was never resumed.

Interviewees perceived that these contextual barriers reduced the possible areas of engagement for the patient partner (e.g., protocol, consent forms, pre-recruitment). Importantly, the efforts required to integrate this embedded study late in the process reduced the buy-in of the host study team. This late integration also led to a continuing perception that the embedded and host studies were two distinct undertakings, led by different teams pursuing different goals.

Another barrier identified was a “culture clash” resulting from divergent visions of engagement with patients, the roles of the respective teams and their ways of conducting research. One member of the host study team pointed to cultural differences between the teams as the biggest problem, as the study involved changing long-standing and deeply-rooted cultures and perceptions.

The host research team’s vision of integrating a patient partner in clinical research appeared to be centered around its instrumental benefit to clinical research, its ability to improve recruitment and retention and its usefulness in obtaining research funding. As one interviewee expressed:

“[Patient involvement] is something now that is really important in the community, and even anything that’s a funder is going to ask us. They’re going to ask us to get the patients more involved as well. That’s generally how it works.” Elisa.

Another interviewee from the host study team indicated that clinical research teams see the patient partner primarily as an additional tool that could facilitate participant retention and similar benefits.

For the embedded study team, their vision of integrating a patient partner in clinical research seemed to focus on the empowerment and humanization of participants’ experience in a clinical trial (i.e., sharing of lived experience, accompaniment of participants, and influence on primary research design):

“I think that the benefits in terms of recruitment and retention should be considered as secondary benefits of the presence of a patient partner […]. You know, it’s the person’s goals first and then, after that, there’s like secondary benefits.” Peter.

These differing perspectives on patient engagement based on the interests, goals and knowledge of both parties contributed to the constant renegotiation of the intervention model. In addition, the utilitarian conception of the patient partner held by the host research team led to a perception of the embedded study team as a service provider, rather than a research team on an equal footing with them.

Contributing to the cultural difference between teams were the language used by the embedded study team and the concrete examples given in training. Host research team members reported that the length (3 h) and content of engagement training (e.g., historical background, engagement in healthcare as opposed to health research) were not adapted to the clinical research context and trial (e.g., content not directly related to clinical trials or not relevant to research nurses participating in the training, limited time of clinical research staff). This communication issue resulted in lower understanding and interest in patient partnership. For example, one interviewee from the host study team commented that the embedded study team used overly domain-specific literary language in its training, and that it needed to be adapted to the reality of everyday practice to make it easier for trainees to understand the benefits of patient partnership.

Some aspects of the research discussed previously can also be considered as barriers, in particular the lack of fit between patients’ needs and the proposed intervention model.

Finally, interviewees reported a sense of burden related to a combination of factors: the imposition of the embedded study by senior researchers, the limited resources to implement the intervention (time, research staff and funds), and the cumbersome process for its integration into the host study.

“Let’s say for the study to exist […], it was the minimum footprint in the [clinical trial] that we could have […]. The only thing that was acceptable was what took the least amount of resources, the least amount of time.” Elisabeth.

Indeed, a member of the host study team stated that they had repeatedly asked for the engagement study to have as little impact as possible on the clinical trial, which the engagement team could have perceived as a sign that the host study team did not want to work with them. This interviewee added that negotiations between teams were demanding work and that meeting every week was impossible.

*Recommendations*. Interviewees offered several recommendations and ideas for future collaboration. The main recommendation was to assess the appropriateness of including patient partners at the outset of a research project, based on the study topic. Once it has been decided to include patient partners in a clinical research project, researchers need to align patient partner follow-up to the needs of study participants by assessing where and how the patient partner can contribute most.

“It has to be a plus to have a patient around the table, compared to being followed by professionals alone. Otherwise, it’s just confusion and then another phone number, and then someone you don’t know what they are for. So the question of health conditions and then needs for the participants, for me, is really key.” Peter.

Interviewees also judged that, to foster a spirit of collaboration and teamwork, it is essential to integrate the teams and the patient engagement component at the design stage of the host and embedded studies. Integrating into a clinical trial that is already underway was seen as detrimental to team cohesion and the implementation of the patient partner intervention. In contrast, working together from the outset would have enabled more discussion and created the conditions necessary to ensure that the engagement component was well integrated with the clinical trial from the start. Early integration would allow for better alignment of goals, values and ways of working together. It would also facilitate the allocation of resources for the recruitment, training and integration of patient partners, including the time of management staff.

Considering the time devoted to inter-team meetings for the embedded study, a member of the embedded study team reflected:

“Then, it was a bit of a, not a shock, but I had to work all the time in a team with teams, sometimes, of eighty or more employees, and then we, we met every week [in other studies], but there [in this study], we met once a month, and each time, we never had time to talk about real issues. I thought, ‘well, we’re not going anywhere,’ so there you go.” David.

Further, interviewees noted the importance of integrating the patient partner into the team at the design stage of the study, as the potential for impact is greater. As one interviewee from the host study team pointed out, as they gain experience with patient partners, a culture shift will occur so that integrating patient partners into clinical trials will require less effort and eventually become the norm.

More generally, it was deemed important to understand the context, language and culture of the research community on both sides. Interviewees recommended finding concrete examples from the literature to establish a common language and vision of patient engagement in clinical research.

Communication specifically was the focus of several recommendations. The first was to use clear, simple, and practical language appropriate to the clinical research context. Interviewees also recommended implementing a recurring communication procedure that includes the patient partner in the loop of exchanges and, for recruitment, implementing direct communication (without intermediaries) between the patient partner and clinical trial participants (rather than via a brochure, an answering machine message and a return call). Interviewees also advised taking advantage of the patient partner’s role in communicating research progress to reach a larger number of participants (e.g., via a newsletter).

Finally, the training on patient engagement provided by the embedded study team to the host research team was the focus of several recommendations. One was to shorten the length of this training to a maximum of 1.5 h by removing unnecessary material. Interviewees also recommended adapting the content to the research context (e.g., clinical, experimental) by adopting language and examples appropriate to this particular context. This would involve focusing the training on the specific project at hand and the expected benefits, for greater buy-in and acceptability.

“The objectives that may be different in each study. I think it is. I think, it’s more the… the objectives, the issues. I think that we can’t just put a framework […] it’s to properly identify our issues, our objectives in relation to the reality.” Lisa.

In addition, an incentive such as continuing education credits could be introduced, and the financial burden of training could be shared by paying for the training time of clinical personnel from research funds. Finally, although a patient partner co-led the training, it was recommended that the patient partner who would be working on the clinical trial be integrated into the training.

## Discussion

This study aimed to investigate the extent to which it is feasible to conduct a trial of patient engagement embedded into a host drug clinical trial. While the attempt at integrating patient partners in an existing drug clinical trial proved challenging on many fronts, it sheds light on key aspects of feasibility to address in the planning, design and implementation of an embedded study of patient engagement in research. Some of those challenges are logistical (e.g., detailed procedures on how to recruit, integrate and collaborate with patient partners), others are regulatory (e.g., adapting existing ethical approvals within the highly regulated standards of drug clinical trials), while others relate to professional, institutional and scientific culture (e.g., challenges to professional identity, differences in scientific epistemologies between clinical trial study teams and participatory research teams).

A comparative analysis of first and final protocols and semi-structured interviews with members of the host and embedded study teams (including patient partners) revealed information on the evolution between the initial and final engagement study design resulting from negotiation between the embedded and host study teams, the feasibility of implementing the engagement intervention, barriers, benefits and recommendations for future embedded engagement studies.

The systematic comparison of protocols was informative on the evolution of the embedded study design over time: compared to the protocol prepared by the embedded study team, the protocol agreed on following months of discussions between the host and embedded study teams was reduced in scope, less integrated into the clinical trial, emphasised objectives of feasibility and acceptability, and provided more predetermined details.

Reflecting on the process of designing and implementing the embedded study, members of both teams judged that feasibility was hindered by an initial reluctance to recognize the role and added value of the patient partner to support clinical trial participants’ follow-up and for study design, and by the nature of the host clinical trial which required relatively little participant support.

Barriers to the implementation of the engagement component include its late integration into the clinical trial, different visions of patient engagement and its potential benefits, differences in communication language style and preferences, a lack of fit between the specific needs of the host study and the proposed engagement model, and an overall sense of burden. However, interviewees perceived that integrating patient partners into the host clinical trial could improve the experience of participants in the host clinical trial through experience sharing, providing support for the consent process and improving knowledge transfer.

Recommendations from interviewees include assessing the appropriateness of including patient partners based on the needs of medical patients in relation to the research topic, integrating patient engagement at the design stage of the host study, developing an understanding of the culture of the host and embedded study teams, clear and regular communication, and finally tailoring the patient engagement training to the clinical trial.

This feasibility study aimed to learn, and both the host and embedded study teams agreed that key points had been learned. For example, barriers to feasibility were identified. From the host study team’s point of view, the study was a work of co-construction, in which both teams were in “solution mode” and found all sorts of ways to adjust the engagement component to existing feasibility constraints to integrate it into the main study. For the embedded study team, one of the key lessons was that, in practice, not all contexts lend themselves to embedded studies. Their understanding of the legal, ethical and organizational framework of a multicenter study was initially limited, which contributed to the difficulty of anchoring the engagement intervention into the real-world operations of an ongoing clinical trial. And perhaps the actual or perceived need for the engagement intervention simply did not exist. Finally, the engagement study was approved by the clinical institution’s ethics review board, which was a first in the province and can be considered a form of success.

It has been observed that very few publications in the engagement literature refer to “negative outcomes and impacts despite their being just as important.” ( [11], p. 15) Such findings can demonstrate how these outcomes come about and how future work can avoid them. Thus, although some of the original objectives of the study were not met (test data exchange procedures, calculate sample size for future clinical trials), the study generated learnings that may nevertheless be useful for future studies. Reflecting on the absence of participants enrolled and the study process more generally, interviewees in our study pointed to the nature of the two studies, the two teams and the timing of the integration between the two studies.

In hindsight, interviewees considered that this particular clinical trial may not have been the most appropriate to offer patient partner support to participants, considering the low to nonexistent side effects of the marketed drug being tested for its preventive effects and the limited perceived added value of having patient partners on the research team. Importantly, interviewees also reflected that patient partner input in the clinical trial overall was greatly limited because the host study was ongoing and thus important decisions had already been made by the time the embedded study team received funding for the project and started working on it. It has been largely advocated in the literature (e.g., [11–15]) that patient input should be sought from the design phase of a study. This paper adds an illustration of the potential barriers to overcome when engagement is attempted later on, and insights into how this may affect the success of patient engagement. Several authors [16–19], and patient involvement has been shown to decrease these concerns by weighing in on the acceptability of study procedures from a patient perspective. In addition, greater interaction between the host research team and patient partners may have led to a change in culture that would have paved the way for further interaction. It is also noteworthy that the latest revision of the World Medical Association’s Declaration of Helsinki on medical research involving human subjects (October 2024; [20]) recognises the need for patient involvement at all stages of research [21], making it an integral part of ethical principles for medical research:

Meaningful engagement with potential and enrolled participants and their communities should occur before, during, and following medical research. Researchers should enable potential and enrolled participants and their communities to share their priorities and values; to participate in research design, implementation, and other relevant activities; and to engage in understanding and disseminating results. [20]

Similarly, the World Health Organization’s recently released “Guidance for best practices for clinical trials” calls for researchers to:

“engage local study participants and communities throughout the research, from an early stage of study design, to ensure that the research addresses questions meaningful to them and adheres to high ethical standards”. [22]

Nevertheless, a greater knowledge of the culture and context of the host research team by the embedded study team may have helped them to encourage greater participation in the co-design of the engagement intervention, if not the clinical trial. It has been suggested that a pathway to impact may be a change in culture following interaction between researchers and patients [11]. When the embedded study team understood that clinical nurses could feel threatened in their professional identity by interacting as colleagues with patients with whom they had an established relationship, the research team was able to address their concerns to some extent, which supports the idea that understanding the professional and institutional culture early on would have helped the process.

Considering the requirement from funders to include patient involvement in research reported by some interviewees in this study and increasingly reported in the literature (e.g., [11, 15, 18, 23]), our results may be particularly relevant to contexts where motivation originates from external pressures. As Gradinger et al. [19] have observed, people bring different values to the involvement of patients and other members of the public in research, which likely influences both the process and impact of research. It cannot be assumed that an interest in engagement will necessarily go hand in hand with a commitment to sharing power and control with patients.

This study is also relevant to explore frequent questions related to the selection of patient partners. Within the team, questions were raised about specific qualifications, competencies and experiences that would be relevant to act as a patient partner in the clinical trial. For some, direct experience of participation in the host study needed to be a core requirement. Others emphasized the importance of more general competencies for building partnerships with research nurses, research team members and study participants, regardless of the specific health conditions of the patient partner. Furthermore, potential ethical issues were raised concerning hiring a patient partner on the research team who was also followed as a patient by his research colleagues.

To maximize the benefits of engagement for all stakeholders, this study suggests that it is critical to understand the values and motivations of all involved so that differences can be managed from the start, as suggested by Gradinger et al. [19] In this study, for example, tailoring engagement training to the host research team’s concerns (e.g., providing scientific evidence of the impact of engagement in clinical trials specifically, making explicit the role and specific contribution of a patient partner as complementary to that of a research nurse, keeping the training short) would likely have facilitated collaboration. Working in a strongly positivist context that values “hard data” from quantitative studies, a key ingredient of success may be to put forward positivist arguments for engagement, even if one considers engagement valuable for entirely different reasons (e.g., democratic values of participation [19]). This could foster buy-in early on, leading to a closer collaboration. In this study, such a strategy could have alleviated tensions related to other aspects of the study (e.g., including patient partners in the co-design of intervention details or not, integrating objectives for all stakeholder groups rather than focusing on objectives valued by researchers, etc.). In a subsequent study, training on patient partnership was changed following recommendations from this study. For example, care was taken to adapt the training to the specific context, its length was reduced to 1.5 h, and a certification of completion was provided to trainees. Several research team members of the clinical institution took this revised version of the training, and their feedback was very positive.

Thus, in the end, despite and partly because of its unintended outcomes, this feasibility study provided valuable lessons for the planning of subsequent clinical trials.

### Strengths and limitations of the study

All interviewees were members of the host or embedded study teams. We addressed this challenge by having interviews conducted by an experienced researcher who had no previous involvement in the study. Another limitation was the relatively small sample (8 interviewees). However, we recruited all individuals who had been actively involved in the integration of the embedded study into the clinical trial, with the exception of one person who had left their employment (a research nurse) and a senior peer mentor who is a coauthor of this article. The final sample included an equal number of interviewees from the host and embedded study teams and represented a diversity of views (researchers, research staff, clinical staff, managers, patient partners).

We had hoped to conduct the study in more than one of the host clinical trial’s sites to implement randomization, but could only conduct the embedded study in a single site to ensure feasibility. Although this can be considered a limitation, the site chosen for the intervention had the largest number of recruited participants for the host study. Considering that no participants were recruited at the largest site in the context of the pandemic, it is unclear that conducting this embedded study at other sites would have generated different learnings.

A strength of this study is its uncovering of the black box of negotiation within a diverse research team to integrate patients as partners within a large clinical trial. Several embedded studies of patient engagement in clinical trials have been published, with a focus on outcomes rather than on the process of implementation [1]. Furthermore, descriptive studies of patient engagement implementation tend to be skewed toward “success stories”, with little insight into the challenges such enterprises naturally entail. Including members of the host and embedded study teams in the reflective process, with an external anthropologist to carry out and analyze the interviews, provides a more comprehensive view of the different perspectives involved.

A limitation to keep in mind is that, by drawing on individual interviews, the analysis may give the impression that barriers and facilitators to patient partnership integration depend essentially on individual or team level factors. An ecosystemic analysis could point toward larger institutional, cultural and systemic factors that may foster or hinder patient partnership in clinical trials. For example, the patient engagement literature emphasizes the importance of flexible, inclusive and iterative design processes, which may be at odds with the strict regulatory environment and scientific culture of clinical trials, where adherence to detailed and predetermined research protocols is considered a cornerstone of research quality.

### Directions for future research

This article raises questions to explore further in future research. One of these questions is the potential ethical and relational implications of a patient changing roles when recruited as a patient partner in research after being a participant in a clinical trial. A related question that merits further inquiry concerns the clinical research nurses’ perception that involving patients as partners in research may be threatening to their professional identity. Work conducted in this area suggests that working in partnership with patients may give rise to tension and resistance, but may also lead to a transformation of health professionals’ identity [24]. Finally, future research could shed light on institutional conditions that can be put in place to foster fruitful cohabitation and collaboration between different and legitimate scientific cultures.

## Conclusions

This study provides a rare glimpse into the implementation of a patient engagement study that encountered several significant barriers. It offers insights into how starting engagement after the design stage can impede the meaningful implementation of an engagement intervention in research. It also identifies the minimal conditions necessary for the successful implementation of an engagement component in a clinical trial. We suggest that these conditions go beyond the often-mentioned resources of time and funds. They include a good level of knowledge of the clinical context from the embedded study team and a commitment from both teams to work together with patients as partners on the co-design of key aspects of the clinical trial. We believe further work into the processes by which trust develops and values evolve in clinical research teams that are first exposed to patient engagement is warranted to reliably create successful conditions for meaningful patient engagement in clinical trials.

## Electronic supplementary material

Below is the link to the electronic supplementary material.

Supplementary Material 1

## Data Availability

The datasets generated and analyzed during the current study are not publicly available due to the authors’ commitment not to disclose the site of the clinical trial, but are available from the corresponding author upon reasonable request.
