# Supplementary material for: Overcoming barriers to implementation of patient engagement in clinical trials: feasibility testing of an embedded study
Source: Res Involv Engagem. 2025 Feb 26;11:15. doi: 10.1186/s40900-025-00689-0 (PMC11866851; doi:10.1186/s40900-025-00689-0)
Supplement: Supplementary file 1 — Supplementary Material 1 [file 40900_2025_689_MOESM1_ESM.docx]

### **Gripp2 Checklist**

| **Section and topic** | **Item** | **Reported on page No** |
| --- | --- | --- |
| 1: Aim  Report the aim of the study | To contribute to the science of engaging patients in clinical trials by addressing the following research question: to what extent is it feasible to conduct a randomized trial of patient engagement embedded into an ongoing multi-center clinical trial? | 2, 6 |
| 2: Methods  Provide a clear description of the methods used for PPI in the study | Two patients were part of the research team and are coauthors. They participated in the design of the study, which included developing and embedding a patient engagement intervention (substudy) into a phase 3 randomized clinical drug trial (host study). The planned patient engagement intervention consisted of discussions between host study participants and a patient partner, to improve research participants’ experience and retention in the trial.  The two patients conducted the recruitment of a third patient to act as patient partner in the engagement intervention. One of the patients co-led the training of the host study team and of the patient partner recruited for the engagement intervention.  Individual semi-structured interviews were carried out with patient partners and other research team members involved in the development and implementation of the substudy, as well as an analysis of project documents. | 2, 6-9, 23, 32 |
| 3: Study results  Outcomes — Report the results of PPI in the study, including both positive and negative outcomes | Patients contributed to the study in several ways, including:   - Providing regular input throughout the study, including contributing to the design of the patient engagement intervention - Conducting the recruitment of a patient partner for the engagement intervention - Co-leading the training of the host study team and of the patient partner recruited for the engagement intervention - Contributing to the paper, by participating in discussions on the draft and reviewing the manuscript. | 7-8, 23, 32 |
| 4: Discussion and conclusions  Outcomes — Comment on the extent to which PPI influenced the study overall. Describe positive and negative effects | Patients were essential to the engagement substudy, as regular members of the research team (paid for their time). They participated in regular research meetings throughout the substudy, and their perspectives were actively sought, valued, and had concrete impact on the study and paper.  However, the integration of the engagement intervention into the host clinical trial faced several challenges. These challenges include the late integration of the engagement intervention into the clinical trial, different visions of patient engagement and its potential benefits, differences in communication style and preferences, a lack of fit between the specific needs of the host study and the proposed engagement model, and an overall sense of burden.  Nevertheless, substudy interviewees thought that integrating patient partners into the host trial could potentially improve the experience of participants in the trial through experience sharing, providing support for the consent process and improving knowledge transfer. | 7-8, 14-21, 24-30, 32 |
| 5: Reflections/critical perspective  Comment critically on the study, reflecting on the things that went well and those that did not, so others can learn from this experience | Patient partners were involved in the engagement substudy as far as possible and this worked well.  The challenges faced to integrate the engagement intervention into the clinical trial led to several reflections. Our key recommendations are:   - Understanding well the context of the clinical trial (e.g., organizational, administrative, regulatory, ethics) - Collaborating to integrate patient engagement at the design stage of the host study - Understanding the values and motivations of all involved so that differences can be managed from the start | 3, 7-8, 21-30, 32 |

*PPI: patient and public involvement*
